# Metabolic endotoxaemia in childhood obesity

**DOI:** 10.1186/s40608-016-0083-7

**Published:** 2016-01-27

**Authors:** Madhusudhan C. Varma, Christine M. Kusminski, Sahar Azharian, Luisa Gilardini, Sudhesh Kumar, Cecilia Invitti, Philip G. McTernan

**Affiliations:** 1 Division of Biomedical sciences,, Warwick Medical School, University of Warwick, UHCW Trust, Clifford Bridge Road, Walsgrave, Coventry CV2 2DX UK; 2 Department of Medical Sciences & Rehabilitation, IRCCS Istituto Auxologico Italiano, Via Ariosto 13, 20145 Milan, Italy

**Keywords:** Endotoxin, Childhood obesity, Cardiovascular injury markers, Insulin resistance

## Abstract

**Background:**

Childhood obesity is associated with chronic low-grade inflammation considered as a precursor to metabolic disease; however, the underlying mechanisms for this remain unclear. Studies in adults have implicated gut derived gram-negative bacterial fragments known as lipopolysaccharide or endotoxin, activating the inflammatory response, whilst the importance in childhood obesity is unclear. The aim of this research is to understand the relationship between circulating endotoxin in childhood obesity, and its’ association with inflammatory and cardiovascular (CV) injury biomarkers.

**Methods:**

Fasted blood was obtained from children with varying degrees of obesity (age: 13.9 ± 2.3Yr; BMI: 35.1 ± 5.2 Kg/m^2^; *n* = 60). Multiplex CVD biomarker immunoassays were used to determine systemic levels of inflammatory and vascular injury biomarkers, such as tumour necrosis factor-α (TNF-α), interleukin (IL-) 1β, 6, 8 and 10, plasminogen activator inhibitor-1 (PAI-1), soluble intercellular adhesion molecule type-1 (sICAM-1), matrix metalloproteinase-9 (MMP-9), myeloperoxidase (MPO) and vascular endothelial growth factor (VEGF) as well as endotoxin levels.

**Results:**

Endotoxin levels demonstrated a significant and positive correlation with the markers for inflammation, vascular injury and atherogenesis (TNF-α: r^2^ = 0.077, *p* < 0.05; PAI-1: r^2^ = 0.215, *p* < 0.01; sICAM-1: r^2^ = 0.159, *p* < 0.01; MMP-9: r^2^ = 0.159, *p* < 0.01; MPO: r^2^ = 0.07, *p* < 0.05; VEGF: r^2^ = 0.161, *p* < 0.01). Males demonstrated significantly higher circulating endotoxin than females (Males: 9.63 ± 5.34 EU/ml; *p* = 0.004; Females: 5.56 ± 4.06 EU/ml; *n* = 60) in these BMI and age-matched cohorts.

**Conclusion:**

The present study demonstrates for the first time a significant association between circulating endotoxin and biomarkers of metabolic risk in children as young as 11 years. Thus, endotoxin-mediated sub-clinical inflammation during childhood obesity may be a key contributor to T2DM and CVD development later in life.

## Background

Environmental, physical and nutritional factors appear critical in determining lifetime disease risk profile for cardiovascular disease (CVD), which is a leading cause of mortality worldwide [1–3]. CVD development in later life shows an increased risk when the condition is preceded by prior chronic inflammatory conditions [4–9]. Therefore the clinical value of determining the factors that induce an inflammatory response in early life appears important to address. Clearly childhood obesity per se has a significant impact on disease risk, inflammation and CVD, however, adipose tissue (AT) may be an early contributor to metabolic dysfunction through prior systemic insults, which initiate an inflammatory response [7, 8, 10–14].

One cellular mechanism for an increased inflammatory response may arise through activation of the innate immune system as observed in human AT [14, 15]. Previous studies have shown that increased activation of the innate immune pathway may arise through excess circulating gut derived bacteria, known as lipopolysaccharide (LPS) or endotoxin; which represents fragments from the outer cell wall membrane of gram-negative bacteria [14–16]. In human AT, it appears endotoxin has an immediate impact on the innate immune pathway, acting via key receptors known as the toll like receptors (TLRs), which recognise antigens such as the LPS component, to initiate an acute phase response to infection [8, 14]. Stimulation of the TLRs leads to intracellular activation of NFκB, a key transcription factor in the inflammatory cascade that regulates the transcription of numerous pro-inflammatory adipocytokines [14, 15]. Therefore whilst in vitro, endotoxin may act as a mediator of inflammation through activation of NFκB, leading to a rapid response, in an in vivo situation that may be further exacerbated by an increasing fat mass, such as in obesity [17–21].

Clinical studies have also implicated gut derived endotoxin as a direct ‘primary mediator’ to activate the inflammatory state, contributing to metabolic disease, with current cross sectional data showing elevated systemic endotoxin levels in conditions of obesity, coronary artery disease, type 2 diabetes mellitus and fatty liver disease [8, 14–16], which is reduced with weight change [17, 21]. Studies in adults has also shown circulating endotoxin to be positively associated with waist, waist-hip-ratio, insulin levels, inflammatory cytokines as well as lipids, including total cholesterol, triglycerides, LDL-cholesterol and negatively associated with HDL-cholesterol [8, 17–21]. Recent studies in obese children and adolescents has demonstrated that systemic inflammatory cytokines such as plasminogen activator inhibitor-1 (PAI-1) and C-reactive protein (CRP) are elevated, along with vascular injury and atherogenesis markers, such as vascular cell adhesion molecule-1 (VCAM-1) and intercellular adhesion molecule type-1 (ICAM-1) [21–24]. Whether such pathogenic biomarkers directly correlate with systemic endotoxin concentrations in obese children and adolescents is undetermined. We have examined and concluded that bactrial endotoxin is a potential biomarker of sub-clinical inflammation and early CVD risk in childhood obesity.

## Methods

### Subjects

A total of 60 (unless stated otherwise in the figure legend) obese children and adolescents with varying degrees of obesity (BMI: mean ± (SD) 35.1 ± 5.2 kg/m^2^; age: 13.9 ± 2.3 years) were recruited among those referred for weight loss intervention to the obesity centre of the Istituto Auxologico Italiano. All subjects were above the age and sex adjusted 97th BMI percentile, which defines obesity according to the Italian BMI charts [25] and had an age range of 8–18 years. The Ethics Committee of the Italian Institute approved this study and informed consent was obtained from all subjects and their parents. All subjects underwent an oral glucose tolerance test (1.75 g/Kg, up to a maximum of 75 g glucose in 250 ml of water) following an overnight fast. Plasma samples were drawn at baseline, after 30 min and 120 min, for determination of plasma glucose and insulin concentration. Categorisation of glucose tolerance status was made using the World Health Organisation criteria [20]. The impaired fasting glucose was defined by fasting glucose levels ≥5.6 mmol/l [26]. Blood samples were drawn for measurement of endotoxin, adiponectin and, markers of inflammation and CVD. Blood pressure measurements were taken as previously described [11]. Insulin resistance was measured by HOMA-IR (fasting insulin x fasting glucose/22.5) [27].

### Biochemical measurements

Serum endotoxin was assayed using a Chromogenic Limulus Amebocyte Lysate (LAL) test, which is a quantitative test for gram-negative bacterial endotoxin (Cambrex, New Jersey, USA) endotoxin-free vials were utilised throughout. Gram-negative bacterial endotoxin catalyzes the activation of a pro-enzyme in the Limulus Amebocyte Lysate (LAL). The initial rate of activation is directly determined by the concentration of endotoxin. The activated enzyme catalyzes the splitting of p-nitroaniline (pNA) from the colourless substrate Ac-lle-Glu-Ala-Arg-pNA. The pNA released was measured photometrically at 405–410 nm following termination of the reaction. The correlation between the absorbance and the endotoxin concentration is linear in the 0.1–1.0 EU/mL range. Intra-assay CV 3 · 9 ± 0 · 46, inter-assay CV 9 · 6 ± 0 · 75. For the purposes of these studies all samples were run in duplicate within the same plate, therefore no inter-assay variability was observed in this study. To assess recovery of endotoxin within the assay, previous studies have utilised known concentrations of recombinant endotoxin (0.25 and 1.00 EU/mL) were added to diluted, pooled plasma to determine whether the expected concentration correlated closely with the actual observed value and whether there were any variations due to reaction with plasma contents [14]. Lyophilized endotoxin (*E. coli* origin) was used to generate a standard curve with the Chromogenic LAL test kit from Cambrex and produced a corresponding curve in accordance with the manufacturer’s instructions. In plasma, the recovery of spiked endotoxin was 82.0 ± 3.3 % efficient, similar recovery data were noted for serum. Plate to plate variability within the same experiment was 7.4 ± 0.9 %, these findings were similar to those observed from assessment by Cambrex [14].

A multiplexed CVD Panel 1 immunoassay (Linco Research, Missouri, USA) was utilised to examine the circulating concentrations of the following inflammatory and CVD risk biomarkers: TNF-α, PAI-1 (tPAI-1, total) CRP, soluble intercellular adhesion molecule type-1 (sICAM-1), soluble vascular cell adhesion molecule-1 (sVCAM-1), MMP-9, MPO, VEGF and soluble endothelial selectin (sE-Selectin). The CVD Panel 1 immunoassay had a sensitivity of 16–50,000 pg/ml for MMP-9, MPO and PAI-1 and further, a sensitivity of 80–250,000 pg/ml for sICAM-1, sVCAM-1 and sE-Selectin; with an intra- and interassay CV of 4.5–12.3 % and 8.5–16.3 %, respectively. Plasma glucose was measured using an automated glucose analyser (Roche Diagnostics, Mannheim, Germany). Serum insulin levels were measured by a chemiluminescent assay (DPC, Los Angeles, USA) with a sensitivity of 14.3 pmol/l and intra- and interassay CV of 3.7 and 6.7 %, respectively.

### Statistical analysis

All analyses were performed using statistical software (SPSS, version 14; Woking, UK). Variables that were not normally distributed were log transformed. Differences between groups were calculated using a Student’s *t*-test for independent samples. A Pearsons’ correlation analysis was used to analyse bivariate relationships between endotoxin and the various markers of inflammation and vascular injury. Data were expressed as mean ± SD. A *p*-value <0.05 was considered statistically significant.

## Results

### Effect of gender on biomarkers of inflammation

From this cohort, biochemical analysis was performed on 24 male subjects and 36 female subjects with matching BMI and age. Clinical and biochemical characteristics of male and female obese subjects are provided in Table 1. No significant gender differences were observed in HOMA-IR, blood pressure or several markers of inflammation and CV injury.

**Table 1:** Clinical and biochemical characteristics for obese, BMI and age-matched male (*n* = 24) and female (*n* = 36) subjects

Clinical and biochemical characteristics	Male	Female	*p* value
	Subjects (±SD)	Subjects (±SD)	
Age (yrs)	14 ± 3	14 ± 2	-
BMI (kg/m^2^)	34.0 ± 5.1	35.6 ± 5.3	-
Fasting Glucose (mmol/l)	4.4 ± 0.3	4.5 ± 0.5	N/S
2 h glucose, mmol/l	6.0 ± 1.0	5.7 ± 1.0	N/S
HOMA-IR	2.6 ± 1.2	3.3 ± 2.1	N/S
Endotoxin (EU/ml)	10.1 ± 5.4	5.3 ± 3.7	p < 0.01**
CRP (μg/ml)	7.5 ± 8.3	9.0 ± 7.5	N/S
TNF-α (pg/ml)	5.0 ± 2.5	5.9 ± 5.8	N/S
PAI-1 (ng/ml)	27.0 ± 10.7	26.0 ± 15.7	N/S
sICAM-1 (ng/ml)	110.4 ± 37.4	103.1 ± 47.9	N/S
Systolic BP (mmHg)	121.0 ± 10.8	119.1 ± 8.5	N/S
Diastolic BP (mmHg)	74.0 ± 11.2	72.7 ± 8.5	N/S

Data are expressed as mean ± SD. significant differences in data between male and female subjects are highlighted (*P*-Value **, *p* < 0.001)

### Correlation of endotoxin with biomarkers of inflammation and CVD in childhood & adolescent obesity

**Fig. 1:**
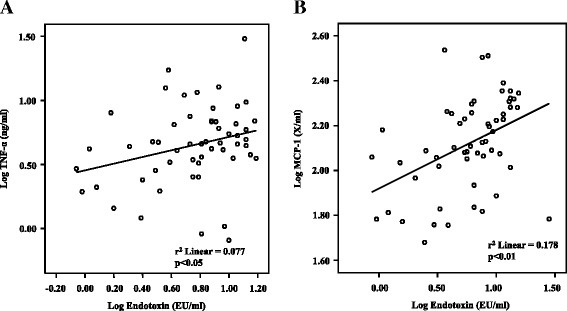
Correlation between log endotoxin (EU/ml) levels and the inflammatory markers: (**a**) log TNF-α pg/ml (*p* < 0.05) (**b**) log MCP-1 ng/ml (*p* < 0.01), in BMI and age-matched children and adolescents

In this study circulating endotoxin concentrations significantly and positively correlated with TNF-α (*p* < 0.05, r^2^ = 0.077) and MCP-1 (*p* < 0.01, r^2^ = 0.178) (Fig. 1a, b). However, no significant correlation was noted between circulating endotoxin levels and CRP (*p* = N.S, r^2^ = −0.069).

**Fig. 2:**
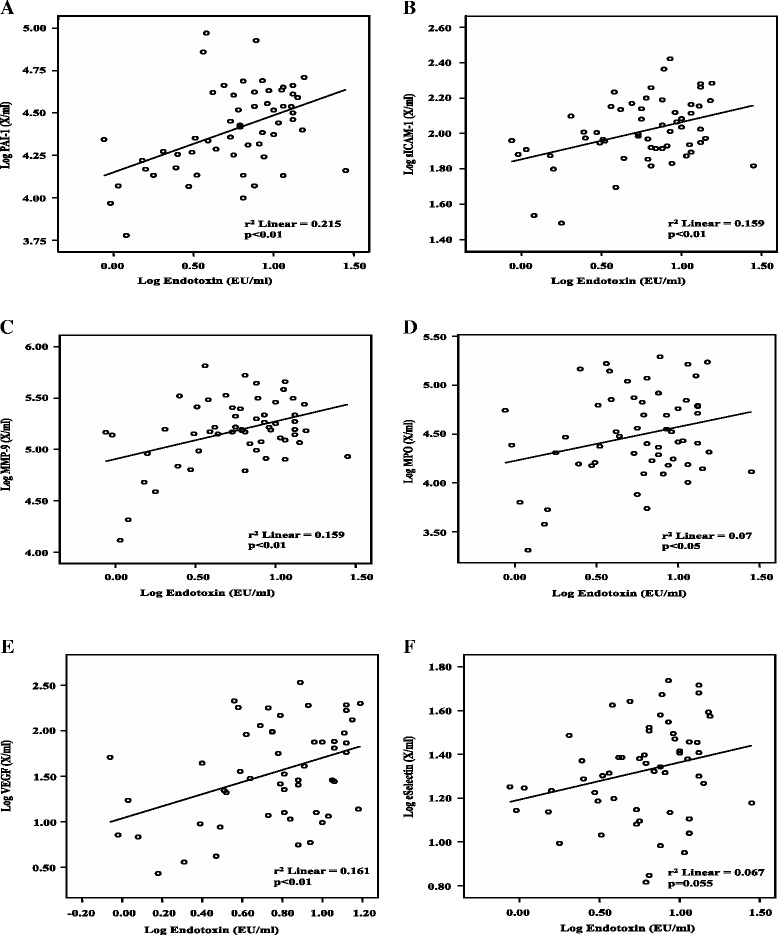
Correlation between log endotoxin (EU/ml) levels and the following markers of CVD: (**a**) log PAI-1 (ng/ml) (*p* < 0.05), (**b**) log sICAM-1 (ng/ml) (*p* < 0.05), (**c**) log MMP-9 (pg/ml) (*p* < 0.05), (**d**) log MPO (pg/ml) (*p* < 0.05), (**e**) log VEGF (pg/ml) (*p* < 0.05) and (**f**) log eSelectin (X/ml) (*p* < 0.05) in BMI and age-matched children and adolescents

With regards to CVD risk markers, circulating endotoxin concentrations further correlated with several parameters of atherogenesis and vascular injury; these included PAI-1 (*p* < 0.01, r^2^ = 0.215), sICAM-1 (*p* < 0.01, r^2^ = 0.159), MMP-9 (*p* < 0.01, r^2^ = 0.159), MPO (*p* < 0.05, r^2^ = 0.07) and VEGF (*p* < 0.01, r^2^ = 0.161) (Fig. 2a–f). No significant correlation was observed between endotoxin and sE-Selectin levels (*p* = 0.055, r^2^ = 0.067) or sVCAM-1 levels (*p* = N.S, r^2^ = 0.054).

**Fig. 3:**
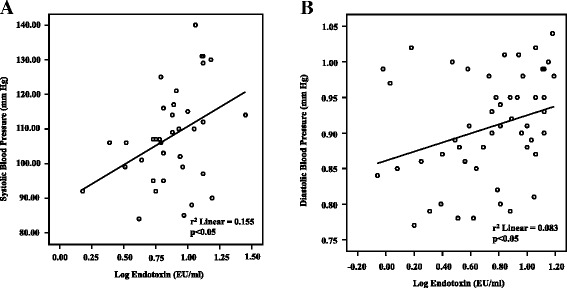
Correlation between log endotoxin (EU/ml) levels and (**a**) systolic blood pressure (mm Hg) (*n* = 37) and (**b**) diastolic blood pressure (mm Hg) (*n* = 51) in BMI and age-matched children and adolescents

**Fig. 4:**
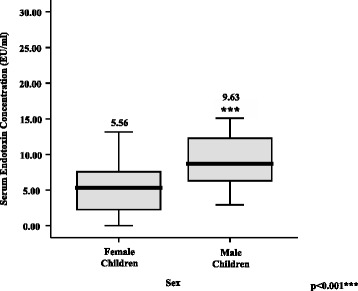
Comparison of the mean relative endotoxin serum concentrations (EU/ml) ± SEM in male and female BMI and age-matched children and adolescents

Additional analysis of serum concentration data revealed that circulating endotoxin levels positively correlated with systolic blood pressure (*p* < 0.05; r^2^ = 0.155; *n* = 37) (Fig. 3a) and diastolic blood pressure (*p* < 0.05; r^2^ = 0.083; *n* = 51) (Fig. 3b). Endotoxin was higher in males than females as a direct comparison (Fig. 4).

## Discussion

These current studies highlight that in childhood obesity circulating endotoxin is significantly correlated with pro-inflammatory markers, TNF-α, MCP-1, as well as biomarkers of atherogenesis and vascular injury including PAI-1, sICAM-1, MMP-9 and MPO, VEGF. Furthermore, data analysis also determined that circulating endotoxin levels are positively correlated with both systolic and diastolic blood pressure in children with obesity. Taken together these findings also indicate that the presence of metabolic endotoxinaemia, which appears to occur in childhood, correlates with pathogenic pro-inflammatory factors in a similar manner to that noted in adults with metabolic disease [14, 15, 17, 21, 22]. These studies also appear to suggest that the raised endotoxin levels in childhood are coupled to a noted high disease risk profile and blood pressure, which together ultimately could lead to an earlier life progression of CVD. Endotoxin may also account for, in part, the continual pro-inflammatory state experienced in obese children [28–31].

These studies also observed for the first time a noted significant rise in circulating endotoxin in boys compared with girls. Whilst this was not the aim of the study these findings appear consistent with gender-specific effects noted in adults; maintained across several ethnicities [19]. Prior studies in adults suggest a higher endotoxin-induced pro-inflammatory cytokine release in men than women, although this study in children did not identify this gender specific aspect [32, 33]. Such a disparity in endotoxin-induced pro-inflammatory cytokine release between the children and adults with obesity may arise, in part, due to the difference in exposure time to endotoxin; which in obese adults may give rise to long-term damaging inflammatory change leading to T2DM [26, 34, 35].

These studies also suggest that changes in inflammation, vascular dysfunction, and blood pressure in childhood obesity may arise beyond the known impact of the cardiometabolic lipid profile [29, 30, 32, 37, 38]. A key mediator to increase disease risk arise from circulating commensal bacterial endotoxin, derived from the gastrointestinal-tract eliciting a pro-inflammatory response in prior childhood and adolescent obesity studies [36, 39, 40]. Additionally, adult studies highlight that diets high in fat and or processed meat appear to raise endotoxin levels further, whilst dairy products and other food combinations may reduce endotoxin levels and inflammation [18, 41–43]. As such further future insight into examining the impact of diet on endotoxin levels in children with obesity may highlight important interventions to reduce the long-term health risk [14, 40, 44–46].

Several risk factors in childhood have been proposed to predict the later development of CVD including obesity, hypertension and endothelial dysfunction promoting atherogenesis and thrombosis [47–50]. In our cohort of obese children and adolescents, several biomarkers of vascular injury and endothelial dysfunction, including PAI-1, sICAM-1, MMP-9, MPO and VEGF, were significantly and positively correlated with circulating endotoxin concentrations. The observed pro-inflammatory biomarker risk profile in the obese children appears to be similar to both what has been identified in adults with CVD as well as studies comparing obese and lean children [50, 51].

Hypertension has been identified as a key risk factor for atherogenesis and vascular injury, and this study noted a positive association between endotoxin with systolic and diastolic blood pressure. As such a diet that raises endotoxin levels would appear to also increase blood pressure. However this is a cross-sectional study, which therefore cannot determine a causal relationship, although previous studies have demonstrated that mice fed a continuous endotoxin bolus, exhibited a subsequent increase in vascular dysfunction [52, 53]. This is consistent with the concept that an endotoxin-induced inflammatory response in childhood obesity, may consequently contribute to an accelerated risk of CVD in later life. Furthermore the noted correlations between circulating endotoxin and biomarkers of endothelial dysfunction, in this childhood obesity study, could also promote a cluster of these pro-atherogenic factors contributing to accelerated atherosclerosis, arterial stiffness and CVD in later life [37, 54–57].

This study had some limitations, namely: the cross-sectional design which did not allow causal determinations and a limited sample size indicating modest correlations. Further, the lack of gender-effects on the correlations between endotoxin and the pro-inflammatory factors may also be considered a reflection of the limited cohort size. Thus, future studies exploring further aspects of these observations in larger cohorts are warranted.

## Conclusion

In summary, this study highlights the relationship between endotoxin and several inflammatory and CVD risk biomarkers, particularly as an early player in obesity-related inflammatory disorders during childhood and adolescence. Furthermore, even at an early age in the obese state, female subjects exhibited lower endotoxin levels than their male counterparts, suggesting a more favourable metabolic profile. This may manifest in later life as delayed CVD mortality and morbidity for females in contrast to males. Finally, among the inflammatory markers evaluated in this study, endotoxin may serve as a potential mediator of sub-clinical inflammation in childhood and adolescent obesity as noted in adult studies.

## Abbreviations

BMI: Body mass index
CRP: c-reactive protein
CV: Cardiovascular
CVD: Cardiovascular disease
HDL: High density lipoprotein
HOMA-IR: Homeostatic model assessment of insulin resistance
ICAM-1: Intercellular adhesion molecule type-1
IL: Interleukin
LDL: Low density lipoprotein
MCP-1: Monocyte chemoattractant protein-1
MMP-9: Matrix metalloproteinase-9
MPO: Myeloperoxidase
PAI-1: Plasminogen activator inhibitor – 1
sICAM-1: Soluble intercellular adhesion molecule type-1
sVCAM-1: Soluble vascular cell adhesion molecule-1
T2DM: Type 2 diabetes mellitus
TLR-4: Toll-like receptors
TNF-α: Tumour necrosis factor-α
VCAM-1: Vascular cell adhesion molecule type-1
VEGF: Vascular endothelial growth factor
